# Modic changes as seen on MRI are associated with nonspecific chronic lower back pain and disability

**DOI:** 10.1186/s13018-023-03839-w

**Published:** 2023-05-12

**Authors:** Lloyd G. Czaplewski, Otis Rimmer, Duncan McHale, Mark Laslett

**Affiliations:** 1 Persica Pharmaceuticals Ltd, 7 Denne Hill Business Centre, Womenswold, Canterbury, Kent, CT4 6HD UK; 2 Veramed Ltd, 5th Floor Regal House, 70 London Road, Twickenham, TW1 3QS UK; 3 Weatherden Ltd, 71 Kingsway, London, WC2B 6ST UK; 4 Mark Laslett, Physiotherapy Specialist, The Sports Clinic, 156 Bealey Ave., Christchurch, 8014 New Zealand

**Keywords:** Modic, Chronic lower back pain, Pain, Disability, Endplate oedema

## Abstract

**Background:**

Estimating the contribution of endplate oedema known as Modic changes to lower back pain (LBP) has been the subject of multiple observational studies and reviews, some of which conclude that the evidence for an association of Modic change with LBP is uncertain while others demonstrate a clear link. The clinical trials demonstrating the benefit of basivertebral nerve ablation, a therapeutic intervention, in a tightly defined homogenous patient group with chronic LBP and Modic changes type 1 or type 2, provides further evidence for the contribution of Modic changes to LBP and shows that in these subjects, nerve ablation substantially reduces pain and disability. These interventional studies provide direct evidence that Modic changes can be associated with lower back pain and disability. This review set out to explore why the literature to date has been conflicting.

**Methods:**

A narrative, forensic, non-systematic literature review of selected articles to investigate why the published literature investigating the association between Modic imaging changes and chronic low back pain is inconsistent.

**Results:**

This review found that previous systematic reviews and meta-analyses included both heterogeneous study designs and diverse patient syndromes resulting in an inconsistent association between Modic changes and nonspecific chronic lower back pain. Re-analysis of literature data focussing on more homogenous patient populations provides clearer evidence that Modic changes are associated with nonspecific chronic lower back pain and that type 1 Modic changes are more painful than type 2.

**Conclusions:**

Studies using tightly defined homogenous patient groups may provide the best test for association between MRI-findings and pain and disability. Clinical benefit of basivertebral nerve ablation observed in randomised controlled trials further supports the association between type 1 and type 2 Modic changes with pain and disability.

## Introduction

The proportion of an adult population seeking hospital care for chronic lower back pain (CLBP) (> 3-months) at least once has been estimated at 0.5% of the population per year [1]. Investment in diagnosis is variable, with guidelines that often do not recommend imaging for patients with axial CLBP, because the results would not change medical practice and the demand for imaging would exceed capacity [2]. With sufficient resources, diagnosis may be possible for > 70% of patients with CLBP [3–5], the rest are characterized as suffering from nonspecific CLBP [6].

Patients with severe and persistent lower back pain and disability have a poor prognosis and respond poorly to conservative therapies [7–9]. Magnetic resonance imaging (MRI) of the lumbar spine may be used to provide an image-based division of nonspecific CLBP patients into subgroups with similar pathologies. Approximately 22% (range 12–37%) of patients with nonspecific CLBP have Modic changes type 1 (MC1) or mixed Modic changes Type 1/Type 2 (MC1/2) [10–16]. Some consider the utility of this division uncertain because the MRI findings are observed in both symptomatic and asymptomatic individuals and may also be related to ageing [17–19]. The pathogenesis of Modic changes (MCs) is still the subject of research with mechano-immunological and infectious pathways, independently or concomitantly involved [20, 21].

A recent systematic review and meta-analysis by Herlin et al*.* identified that there was no strong association between lumbar MRI findings and pain or disability in patients with CLBP, because of the heterogeneity of the published studies and of the clinical outcome measures used. They recommended new studies to evaluate the association of MCs with LBP and supported current guidelines restricting the imaging of patients with nonspecific CLBP [22].

Until now, the field has been largely observational. With the observed clinical benefit in randomised controlled trials of basivertebral nerve ablation (BVNA) and its subsequent regulatory approval for the treatment of patients with MC1 and MC2 endplate oedema and vertebrogenic pain, the importance of identifying Modic changes has been recognised [5, 23–26]. BVNA treatment can provide clinically significant reductions in pain and disability [27]. The contribution of vertebral endplate and marrow damage, observed as MC1 or MC2 changes on MRI, to CLBP is becoming more widely appreciated and has led to the endorsement of the diagnostic selection of CLBP patients with MC1 or MC2 changes and the BVNA procedure by US learned societies [23, 24]. The medical history and MRI-based diagnosis of vertebrogenic pain has been recognised with an International Classification of Diseases (10th Revision) diagnostic code M54-51 [28].

It is important to reassess past literature now that the evidence for the clinical importance of recognising MCs is clearer, to understand why observational and interventional studies might have reached different conclusions.

We have conducted an independent evaluation of the analyses conducted in the Herlin et al. review in order to investigate if reducing the heterogeneity of the studies altered the strength of the association between Modic changes and chronic low back pain. In addition, we have conducted a review of the studies published since Herlin et al. We have focussed on studies exploring MRI findings and MCs and their association with CLBP and disability, in well-defined more homogenous populations of patients with nonspecific CLBP.

## Methods

Articles cited in the systematic review/meta-analysis by Herlin et al*.* were inspected and assessed against the Herlin et al*.* inclusion/exclusion criteria [22]. Publications that met all of the Herlin et al*.* criteria were included and the outcomes were then re-evaluated. This led to the removal of some studies which Herlin et al*.* included in their analysis. The methodology in source publications was inspected in detail and, where possible estimates were recalculated to reduce heterogeneity in the populations, e.g. where a publication focussed on pain associated with Modic changes type 1 (MC1) compared to non-MC1, the latter being defined as Non-Modic plus Modic changes type 2 (MC2) and Modic changes type 3 (MC3), a more meaningful comparison of MC1 versus non-Modic (MC0) was calculated. MC2 versus MC0 and MC3 versus MC0 were also calculated. All statistical calculations were performed using SAS™ 9.4 software [29]. Aggregated unadjusted odds ratios were obtained using a Cochrane Mantel-Haenzel (CMH) estimator.

Papers citing Herlin et al*.* were identified using PubMed [23, 30–49]. The literature was searched through PubMed with the keywords “MRI (back pain) association Modic” restricted to the last 5 years and identified 135 articles. The search date was 26 November 2022. Inspection of the abstracts identified 75 papers of potential relevance which were investigated in detail. Papers were included in this review if they had a good clinical question as their basis, identified useful patient subgroups, e.g. acute versus chronic LBP, mild versus severe LBP, excluded confounding diagnoses to provide a clean, nonspecific CLBP data set, MCs were reported by type and reported pain and/or disability. Suitable papers were included regardless of whether they reported an association of Modic changes with pain and disability or not.

## Results

Herlin et al*.* found that six of 13 studies reported a statistically significant association of MC1 and LBP [50–55] and seven reported nonsignificant associations [12, 56–61]. This review of Herlin et al*.* and the source publications cited, focussed on studies with a homogenous and appropriate patient population. Seven of the 13 articles were not considered relevant to our study as they were investigating the interaction between MCs and the efficacy of facet joint injections [12], patients with scoliosis [50], focussed on herniation [56], included acute LBP, and subjects with current herniation [54, 55, 57] or were paediatric cases of uncertain relevance for adult CLBP [60]. One article focussed on nonspecific CLBP subjects, but was a small case-controlled study [61].

Five of the 13 articles selected by Herlin et al*.* investigated the relationship between concordant pain on provocative discography and defined types of MCs [51–53, 58, 59]. Four of these studies selected nonspecific CLBP subjects. Thompson et al. included 11.8% of subjects with current herniation [53]. The studies by Weishhaupt et al. [51], and Thompson et al. compared MC1 versus MC0 + MC2 + MC3, and MC2 versus MC0 + MC1 + MC3. As MC1, MC2 and MC3 can be painful, this comparison weakens any difference. Therefore, the comparisons of MC1, MC2 and MC3 with MC0 were calculated. The 2 × 2 tables for each publication are presented with pain identified by controlled provocation discography as ‘disease’ and MCs as index test (Table 1), the Odds Ratios (Tables 2, 3 and 4) and the properties of provocative discography as a diagnostic test for Modic changes in Table 5.

**Table 1:** 2 × 2 tables for the studies used to calculate Odds Ratios and diagnostic test parameters: Discography provoked Pain as ‘disease’ and Modic changes as Index test

Study		Pain	No pain		Pain	no pain		Pain	No pain
Braithwaite [58]	MC1	5	0	MC2	16	2	MC3	3	0
	MC0	69	60	MC0	69	60	MC0	69	60
Thompson [53]	MC1	126	29	MC2	81	45	MC3	12	9
	MC0	641	1504	MC0	641	1504	MC0	641	1504
O’Neill [52]	MC1	15	2	MC2	18	2		–	–
	MC0	206	217	MC0	206	217		–	–
Weishaupt [51]	MC1	14	2	MC2	9	1		–	–
	MC0	25	65	MC0	25	65		–	–
Kokkonen [59]	MC1	7	10	MC2	7	9	MC3	1	1
	MC0	22	35	MC0	22	35	MC0	22	35

Note Pain and No Pain refer to positive and negative provocation discography, the reference standard. MC0 = no Modic Changes, MC1 = Type 1 Modic Changes, MC2 = Type 2 Modic Changes, MC3 = Type 3 Modic Changes. These are the index tests. Thompson et al. included 11.8% of subjects with current herniation

**Table 2:** Revised association of MC1 with pain on provocative discography

Study	MC1 association with pain on provocation discography OR (95% CI)	
	Herlin et al.	This review
Braithwaite [58]	9.58 (0.52, 176.75)	9.58 (0.52, 176.75)
Thompson [53]	9.32 (6.17, 14.09)	10.19 (6.74, 15.42)
O’Neill [52]	7.90 (1.79, 34.97)	7.90 (1.78, 34.97)
Weishaupt [51]	13.59 (2.92, 63.28)	18.20 (3.86, 85.90)
Kokkonen [59]	1.34 (0.48, 4.00)	1.11 (0.37, 3.36)
Aggregated MC1 OR	6.14 (2.47, 15.27)	8.34 (5.86, 11.87)

**Table 3:** Revised association of MC2 with pain on provocative discography

Study	MC2 association with pain on provocation discography OR (95% CI)	
	Herlin et al.	This review
Braithwaite [58]	6.96 (1.54, 31.50)	6.96 (1.54, 31.49)
Thompson [53]	0.90 (0.62, 1.31)	4.22 (2.90, 6.15)
O’Neill [52]	9.48 (2.17, 41.37)	9.48 (2.17, 41.37)
Weishaupt [51]	15.46 (1.89, 126.67)	23.40 (2.82, 194.34)
Kokkonen [59]	1.03 (0.36, 2.95)	1.24 (0.40, 3.80)
Aggregated MC2 OR	3.15 (1.00, 9.93)	4.46 (3.22, 6.18)

**Table 4:** Revised association of MC3 with pain on provocative discography

Study	MC3 association with pain on provocative discography OR (95% CI)	
	Herlin et al	This review
Braithwaite [58]	6.06 (0.31, 120.35)	6.09 (0.31, 120.35)
Thompson [53]	2.51 (1.05, 5.97)	3.13 (1.31, 7.46)
Kokkonen [59]	–	1.59 (0.09, 26.76)
Aggregated MC3 OR	–	3.39 (1.50, 7.64)

Note that in this study the estimates of the OR of the articles are similar but not identical to those reported by Herlin et al*.* For individual studies where no discs are reported for a combination of Modic type and pain/no pain, a correction of 0.5 was added to all cells before calculating the OR in this review. For the calculation of the aggregated OR, this correction is not used. Finally, for Kokkonen et al. [30] discs recorded with indifferent pain are not included in the pain/no pain categories

**Table 5:** Diagnostic accuracy of Modic changes to pain for individual studies

Study	Statistics	MC1 versus MC0	MC2 versus MC0	MC3 versus MC0
Braithwaite [58]	Sensitivity (%)	6.76	18.82	4.17
	Specificity (%)	100.00	96.77	100.00
	PLR	–	5.84	–
	NLR	0.93	0.84	0.96
Thompson [53]	Sensitivity (%)	16.43	11.22	1.84
	Specificity (%)	98.11	97.09	99.41
	PLR	8.68	3.86	3.09
	NLR	0.85	0.91	0.99
O’Neill [52]	Sensitivity (%)	6.79	8.04	
	Specificity (%)	99.09	99.09	
	PLR	7.43	8.80	
	NLR	0.94	0.93	
Weishaupt [51]	Sensitivity (%)	35.90	26.47	
	Specificity (%)	97.01	98.48	
	PLR	12.03	17.47	
	NLR	0.66	0.75	
Kokkoken [59]	Sensitivity (%)	24.14	24.14	4.35
	Specificity (%)	77.78	79.55	97.22
	PLR	1.09	1.18	1.57
	NLR	0.98	0.95	0.98

For these diagnostic statistic calculations, Pain is used as the reference test

*PLR* Positive likelihood ratio, *NPL* Negative likelihood ratio

**Fig. 1:**
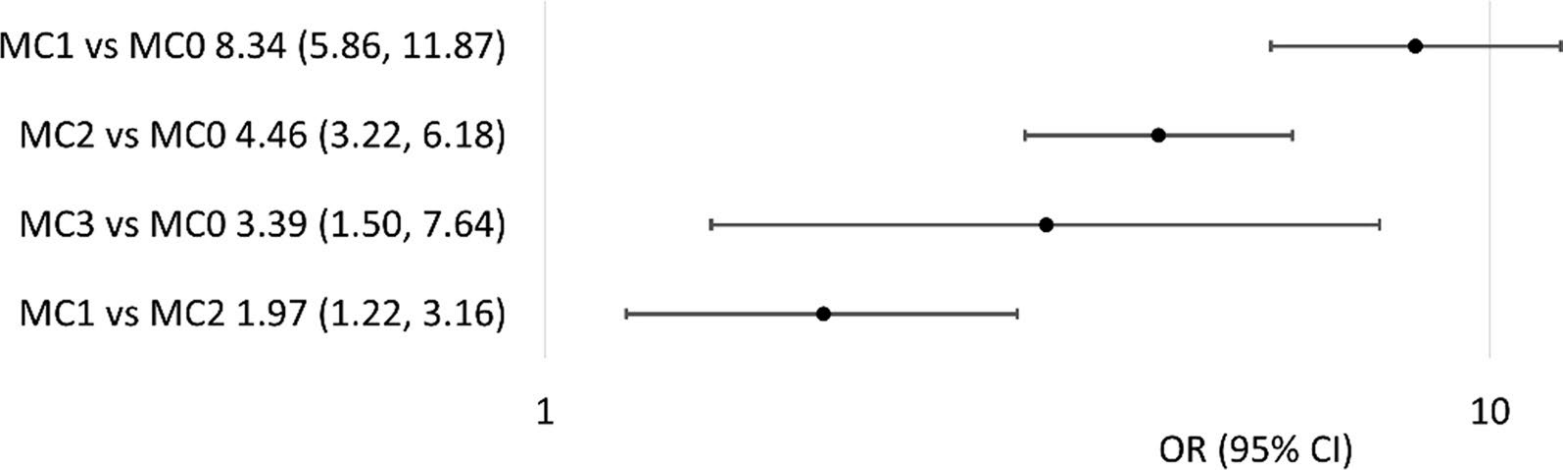
Forest Plot of Aggregated Odds Ratios (95% CI) for pain on provocative discography. Odds ratios and confidence intervals are plotted on a log_10_ scale axis. *CI* Confidence interval, *OR* Odds Ratio

Focussing on non-specific CLBP and recalculating the association estimates used by Weishaupt et al. and Thompson et al., a clearer association between chronic low back pain and Modic changes becomes apparent: Five articles explored whether MC1 and MC2 were associated with discography-induced pain in adult patients with CLBP [51–53, 58, 59]. For MC1, three of the studies found a significant association with pain on discography, and two studies found a non-significant association (Table 2). The non-significant studies had the lowest number of Modic subjects and in one of them the OR was very similar to the 3 positive studies but the small sample size results in wide confidence intervals and a lack of statistical significance. For MC2, four of the studies found a significant association with discography-induced pain, and one study found a non-significant association (Table 3). For MC3, one of the three studies, the largest, found a significant association with discography pain (Table 4). Although Herlin et al*.* summarised Braithwaite et al*.,* as a nonsignificant association, a significant association of any MC, MC2, and MC3 with pain on provocative discography was observed in this study. As the five studies were of similar design, the aggregation of the data using the CMH estimator was performed and resulted in the following results: for MC1 versus MC0, an OR of 8.34 (5.86, 11.87), for MC2 versus MC0 an OR of 4.46 (3.22, 6.18) and for MC3 versus MC0 an OR of 3.39 (1.50, 7.64) was estimated for pain on provocative discography (Fig. 1). The OR for MC1 versus MC2 pain on provocative discography is 1.97 (1.22, 3.16).

The analysis conducted by Herlin et al. suggested that discs associated with MC1 and MC2 have 6.14 and 3.15 times higher odds of pain on provocative discography than MC0 and that there was no statistically significant difference between MC1 and MC2. Reanalysis of the data demonstrates the same numerical trend but with an increase in differentiation between MC1 and MC2 due to the recalculation comparing MC type with MC0. Our analysis demonstrated that MC1 and MC2 have 8.34 and 4.46 times higher odds of pain on provocative discography than a disc not associated with MCs, and that there is a significant difference between MC1 and MC2, with MC1 1.97 times higher odds of pain on discography than MC2 (Fig. 1).

Tables 1, 2, 3 and 4 provide diagnostic accuracy data for MC1, MC2 and MC3 in relation to the criterion standard of discogenic pain on provocative discography. In general, our analysis correlates well with that of Herlin et al. but the signal size is greater. For clinical utility, a high level of specificity, that is, a low false positive rate, is diagnostically relevant (Table 5). MC1 or MC2 seen on MRI indicates a high probability of anterior column pain, as would be confirmed by provocation discography.

Herlin et al*.* considered six articles when concluding that LBP is the same with and without MCs [12, 56, 57, 62–64]. Considering adult nonspecific CLBP without current herniation and excluding confounding pain generators e.g. facet joint degeneration, etc., only Kleinstruck et al. [63] may be relevant, but they did not differentiate between MC1 and MC2, aggregating the MC data. Our reanalysis demonstrating a significant difference between MC1 and MC2 would suggest it is more appropriate to treat them separately. Furthermore, Kleinstruck et al*.* excluded subjects with constant or persistent severe pain and selected a biased patient group with modest pain scores. Herlin et al*.* did not identify any relevant articles assessing whether LBP is different between MC1 and MC2 in adult patients with severe and disabling nonspecific CLBP.

Herlin et al*.* reported seven studies characterising activity limitations and MC [55, 56, 61, 63–66]. Excluding studies on herniation [55, 56, 64], young gymnasts, not representative of the typical CLBP patient population [66], LBP not CLBP [65] and those that did not differentiate between MC1 and MC2 [63]; leaves the study by Rannou et al. [61]. This study only included 36 patients. 12 each with MC0, MC1 and MC2. Patients with MC1 patients reported more pain during the night and in the morning than those without MCs or with MC2s, *p* = 0.001 and *p* = 0.002, respectively.

The articles published in the last 5 years that reported associations between MRI findings and Modic changes in patients with back pain were reviewed to evaluate the current state of the art. Two reviews and four primary studies of relevance were identified. In addition, articles on basivertebral nerve ablation to treat CLBP patients with Modic 1 or Modic 2 changes were identified.

Hopayian et al*.* recognised that previous systematic reviews of the association of MC with LBP have methodological flaws and set out to provide a comprehensive authoritative review [67]. As with the Herlin et al*.* meta-analysis, Hopayian et al*.,* found that inclusion of heterogeneous studies confounded their review. The heterogeneity precluded a meta-analysis and they provided a narrative review instead. Interestingly, they conclude that no conclusions could be drawn from their review, but then say that clinicians should not look for the presence or absence of MC to guide their treatment of patients with LBP. In the absence of an approved treatment for LBP associated with Modic changes this recommendation may have been valid but needs now to be reassessed given the results of BVNA over the last 5 years.

Lambrechts et al*.* performed a systematic review and meta-analysis to assess whether MCs affect surgical outcomes of the cervical and lumbar spine, concluding that they did not [30]. But the patients included in these studies were mostly treated for an acute herniation event [68–71].

Kasch et al*.* introduce their study as a focus on non-specific LBP but then provide a dataset on a diverse cohort and included both acute and chronic LBP and subjects with specific causes of LBP, e.g. herniation and Schmorl’s modes, comparing MRI and baseline findings with clinical outcomes at 6-years. They concluded that MRI degenerative findings at baseline do not have clinically important associations with low back pain [72]. Subjects with LBP at baseline, were of modest severity and disability. The study used a custom questionnaire with a long 3-month recall and composite endpoints that do not allow comparison with other studies. The authors may have set an unrealistic hurdle for clinically important improvement in patients with mild symptoms and commented that no tested associations lead to a difference of more than one unit on their LBP severity scale. A one-unit difference would have required a ≥ 35% change from the mean, which may be impossible in patients with only mild symptoms. Our review suggests that a sub-analysis focussing on the subjects with Modic changes may have been informative.

Çevik et al*.*, report a retrospective case-controlled study of 129 subjects with nonspecific CLBP that met strict criteria [73]. Subjects with MC1 + MC1/2 reported significantly more pain at baseline (+ 29.3%) and at 13-months (+ 25.7%) than subjects without MCs. This study confirms the findings of Jensen et al. [74].

Korhonen et al*.* selected a homogenous patient population with axial nonspecific CLBP (> 6-months) that did not respond to conservative therapy with sufficiently severe disability to consider surgical intervention [75]. The diagnosis of discogenic LBP was achieved using discoblock (intradiscal lidocaine) to transiently relieve pain rather than provocative discography to induce pain. Non-MC mean reduction in LBP NRS pain was − 2.5, MC1 − 5.0, and MC2 − 4.0. The pain reductions in subjects with MC1 were significant (*p* = 0.012) and they concluded that MC1 are associated with lumbar spinal pain with the anaesthetic injection that relieves both disc and endplate pain. The major limitation of the study was its size, as it was too small to be definitive, but the data would fit with the findings in our analysis and with the literature on BVNA.

Udby et al*.* in a retrospective cross-sectional observational study found that the size of MC may have more impact on pain, disability and health-related quality of life than the type of change, but the study was not large enough to allow comparison of small, medium and large MC1 and MC2 [76].

Dimitriou et al*.* [77] investigated the interactions between facet joint degeneration and MCs, finding that facet joint infiltration was significantly more effective as assessed by LBP NRS in patients without MCs compared to those with MCs. However, this was a relatively small study compared with Bianchi et al. 42 versus 226 subjects, which concluded there was no difference [12].

In a comprehensive editorial in Pain Medicine, Conger et al*.* provide a seminal review of the identification and treatment of patients with axial nonspecific low back pain with basivertebral nerve ablation [5]. Patients most likely to respond to BVNA present with inflammatory symptoms of night pain, greatest pain in the morning, prolonged morning stiffness, and pain exacerbation with activity. On MR imaging, radiological findings include endplate bone oedema at the painful level characterised as MC1 or MC2 changes. BVNA treatment provided lasting and clinically significant reductions in pain and disability, with 64% (95% CI 43–82%) and 75% (95% CI 63–85%) of subjects reporting ≥ 50% pain reduction and ≥ 15-point reduction in Oswestry Disability Index at 12 months [27]. Patients with nonspecific CLBP can present with multiple MRI findings, but only the presence of axial lower back pain and MC1 or MC2 changes was predictive of the success of BVNA [23].

## Discussion

Herlin et al*.*, intended to only include studies of nonspecific LBP of any duration and excluded specific causes of LBP and post-surgical patients in their review. Unfortunately, the inclusion of a more heterogenous patient population than intended resulted in inconclusive results which they acknowledged. This increased heterogeneity resulted from: including publications that grouped all types of MC together; not differentiating between acute and chronic LBP; including patients with current herniation or scoliosis. Herlin et al*.* acknowledged that the heterogeneity of studies included was a problem but rather than reduce the heterogeneity they included all of the studies which resulted in a confounded analysis. We do recognise that having published their review protocol in Prospero they had limited freedom to do so. Unfortunately, Herlin et al*.* is often being inappropriately cited as demonstrating no association between LBP and MC without acknowledging its limitations [30–38], but others do cite it in a more appropriate nuanced way [23, 39–49].

When strict criteria are used and a more homogenous patient population is evaluated, a clear association between MC1 and MC2 with pain on provocative discography and subject pain and disability can be observed [47–51]. This homogenous patient group also respond to BVNA and Discoblock procedures [5, 75]. Herlin et al., recommended new studies to assess the association of MRI-findings with pain and disability. We agree and propose that future studies should include designs specifically focussing on homogenous patient subgroups that are difficult to treat, e.g. severe and persistent nonspecific CLBP, which may aid understanding of the underlying pathologies and provide additional options for treatment.

Understanding the relationships between the disc, endplate, and vertebral marrow in health and disease and their relationship with pain generation, perception and disability is complex and multidisciplinary [78, 79]. There is a substantial body of work suggesting that the pathologies underlying MCs have an impact on patient pain and disability [5, 80–82]. The magnitude of effect may depend on patient selection, psychosocial factors and characterisation of MCs, as now evidenced by the efficacy of BVNA.

Disc degeneration can be initiated by endplate damage or disc herniation, leading to a cascade of inflammatory activities that result in MCs [78, 83, 84]. Taken together, vertebrae with oedema of the endplate and marrow with a degenerate disc are very likely to be painful, and the pain may be vertebrogenic or discogenic or both. The spinal level at which MCs are found and variation in the extent of endplate involvement, the type, size, and intensity of oedema, and the degree of disc degeneration can all influence the severity of pain and disability [85]. This complexity cannot be captured with simple clinical trial protocols, which average the number of MRI-findings across a heterogeneous group of subjects and time. The use of homogeneous subject groups without confounding pain generators may be required to adequately test aetiologic hypotheses and to define biological processes.

With the approval of BVNA to treat vertebrogenic chronic low back pain, there is now a therapeutic intervention available to treat patients with MCs at a painful level, but it requires magnetic resonance imaging for diagnosis of vertebrogenic pain. The imaging guidelines, which currently recommend that these patients should not be imaged, will need to be reviewed in response to the changing clinical options for these patients and will almost certainly lead to an increased use of imaging for nonspecific CLBP patients.

The analyses to date assume that each MC type represents a homogeneous condition and underlying disease process but even within these sub-groups there are additional factors including both biological and psychosocial factors which can influence the pain and disability experienced by patients. Modic changes can be associated with pain and disability, pain on provocative discography, and in a proportion of subjects, can be asymptomatic. What is not clear is whether the painful and asymptomatic MCs are the same. Population surveys of asymptomatic subjects can find up to 13.5% MC1 and 25.4% MC2 [16]. In the five provocative discography studies (Table 1), reporting the median and the range of proportion of cases with no pain on provocative discography across the studies, MC1 was not painful on provocative discography in 12.5% (range 0–58.82%) and MC2 not painful on provocative discography in 11.1% (range 10.00–56.25%) of the cases. The observation of this level of asymptomatic MC is enough to create confusion in the literature. It is not known whether nonpainful MC may develop into painful MCs, whether concordant pain on discography is consistent over time, or whether the basivertebral nerve may be damaged in some subjects. Research into the reasons behind painful and non-painful MCs is needed.

Some limitations must be taken into consideration when interpreting the results of this study. One of the authors (LGC) works for Persica Pharmaceuticals Ltd, and two authors (OR and DM) work on a Persica sponsored clinical trial investigating an intradiscal antibiotic to treat patients with CLBP and Modic type 1 changes and therefore disclose an interest in Modic changes being associated with pain and disability and we acknowledge that this may introduce a sub-conscious bias. LGC provided the literature review and if any sub-conscious bias was introduced it would most likely be at this stage of the review process. This was not a systematic review or meta-analysis and a review protocol was not prospectively made public. This review proposes that the effects of MRI-findings on pain and disability should be studied in defined homogenous sub-groups of patients to isolate that finding and to minimise confounding causes of pain. This reductionist approach may enable MRI-findings to be investigated in specific patient groups, but the findings may not be generalisable to subjects with multiple findings and more complex disease.

We conclude that Modic changes type 1 and type 2 can be painful, and that the confusing literature is due at least in part to heterogeneous study selection, inclusion of patients with a diversity of syndromes and inappropriate reporting. Patients with debilitating CLBP and Modic changes may be denied effective therapy if the current literature continues to be cited and inappropriate conclusions drawn. Basivertebral nerve ablation may be effective, but it is an invasive, irreversible procedure. Whether it becomes widely adopted or not, it provides an intervention which indicates that Modic changes type 1 and 2 do contribute to pain and disability that can be alleviated. Future alternative treatments that address the causes of Modic changes such as inflammation and infection, or the consequences of Modic changes such as bone remodelling, may find clinical utility and offer patients and physicians greater choice of treatment options.

## Data Availability

Not applicable.
